# The adequacy of policy responses to the treatment needs of South Africans living with HIV (1999-2008): a case study

**DOI:** 10.1186/1758-2652-12-37

**Published:** 2009-12-14

**Authors:** Jeff A Gow

**Affiliations:** 1 School of Accounting, Economics and Finance, University of Southern Queensland, Toowoomba, Australia; 2 Health Economics and HIV/AIDS Research Division (HEARD), University of KwaZulu-Natal, Durban, South Africa

## Abstract

**Introduction:**

South Africa has the largest HIV/AIDS epidemic of any country in the world.

**Case description:**

National antiretroviral therapy (ART) policy is examined over the period of 1999 to 2008, which coincided with the government of President Thabo Mbeki and his Minister of Health, Dr Manto Tshabalala-Msimang. The movement towards a national ART programme in South Africa was an ambitious undertaking, the likes of which had not been contemplated before in public health in Africa.

**Discussion and evaluation:**

One million AIDS-ill individuals were targeted to be enrolled in the ART programme by 2007/08. Fewer than 50% of eligible individuals were enrolled. This failure resulted from lack of political commitment and inadequate public health system capacity. The human and economic costs of this failure are large and sobering.

**Conclusions:**

The total lost benefits of ART not reaching the people who need it are estimated at 3.8 million life years for the period, 2000 to 2005. The economic cost of those lost life years over this period has been estimated at more than US$15 billion.

## Introduction

South Africa is the epicentre of the HIV/AIDS epidemic that is severely affecting nearly all countries in sub-Saharan Africa. In 2008, the Joint United Nations Programme on HIV/AIDS (UNAIDS) estimated that South Africa has the highest number of HIV-positive individuals in the world, with the number of people living with HIV totalling 5,700,000 (CI: 4.9 million-6.6 million). The prevalence rate for adults aged 15 to 49 is estimated at 18.1% (CI: 15.4%-20.9%), and the number of adults aged 15 and older living with HIV at 5.4 million (CI: 4.7 million-6.2 million). Women aged 15 and older living with HIV are disproportionally affected: the figure totals 3.2 million (CI: 2.8 million-3.7 million). The number of children aged up to 14 living with HIV totals 280,000 (CI: 230,000-320,000). And the number of deaths due to AIDS in 2007 was 350,000 (CI: 270,000-420,000) [1]. The raw data on the human impact of the epidemic in terms of ill and dying people is frightening, even in a country with a population of 47 million people.

Prior to 1990, the level of HIV/AIDS infection in South Africa was relatively insignificant (less than 1%). The issue of major national importance was the struggle to obtain democratic freedoms, which the majority of citizens were denied by the apartheid governments of the (white) National Party of South Africa. Democracy came in 1994 with the election of Nelson Mandela as the first freely elected President. Significantly, his deputy was Thabo Mbeki, who took over from Mandela subsequent to the 1999 election and continued in the role of President until 2008.

Dr Manto Tshabalala-Msimang was appointed Minister of Health in 1999 and had responsibility for health policy, including HIV/AIDS and she continued in that role until 2008. She was a strong political ally of President Mbeki throughout this period.

This paper undertakes an assessment of the response of the South African Government to the epidemic over the period, 1999 to 2008. It focuses on one of the most important issues of the epidemic, namely, access to treatment with antiretrovirals (ARVs) in an attempt to explain the efficacy of policies and programmes implemented to address the social, political and economic challenges that widespread and high levels of untreated HIV pose for nations.

## Case description

### National Strategic Plan 2000-2005

The first substantive policy action by President Mbeki's government was instigating a national consultative process with the aim of developing a National Strategic Plan (NSP) for HIV/AIDS and sexually transmitted infections (STIs) in 1999. A National AIDS Council was set up to oversee these developments. The NSP 2000-2005 was a rather thin 31-page document, which had four priority areas and attached goals [2]. The treatment priority had three goals: to provide treatment, care and support services in health facilities; to provide adequate treatment, care and support services in communities; and to develop and expand the provision of care to children and orphans. To achieve these treatment priorities, five strategies were identified:

1. Develop guidelines for the treatment and care of HIV/AIDS patients in health facilities and the community.

2. Ensure uninterrupted supply of appropriate drugs for the treatment of opportunistic infections and other related conditions.

3. Build capacity of health professionals to provide comprehensive HIV/AIDS, STI and tuberculosis (TB) treatment, care and support.

4. Establish strong links between health facilities and community-based support programmes.

5. Improve prevention and treatment of TB and other opportunistic infections.

Many in civil society perceived the plan as inadequate and timid in its responses, particularly given the lack of financial commitment to achieve the rather modest goals. The Treatment Action Campaign (TAC) was at the forefront of agitating for more resources to be pledged for HIV overall and treatment in particular. AIDS advocates, particularly the TAC, campaigned for a programme to use ARVs for prevention of mother to child transmission (PMTCT), and then for an overall national treatment programme for AIDS that included making ARVs accessible.

### Operational Plan for Comprehensive HIV/AIDS Care, Management and Treatment 2003

In July 2002, government established a Joint Health and Treasury Task Team to investigate issues relating to the financing of an enhanced response to HIV/AIDS, based on the NSP 2000-2005. A particular focus of the task team was the treatment component of the NSP, namely, treatment, care and support for those infected and affected by HIV and AIDS.

As a result of much political pressure and agitation, in November 2003, the Mbeki government approved the operational plan that provided the structure for a comprehensive response to HIV and AIDS, including a planned national rollout of antiretroviral therapy (ART) to all South Africans and a PMTCT programme, both through the public health system. Until 2003, South Africans with HIV who used the public health system could get treatment for opportunistic infections they suffered because of their weakened immune systems, but could not get ART, designed to specifically target HIV. The plan was ambitious and projected to cost 11.986 billion South African rands over five years.

The comprehensive plan included the following characteristics [3]:

1. Development of provincial implementation plans to be based on the district health systems within each province.

2. Procurement and/or production of necessary medications and consumables at the lowest prices possible.

3. Upgrading of the national health laboratory system to handle a significant increase in diagnostic testing and monitoring of patient safety.

4. Elaborating an integrated nutritional programme for people living with HIV and AIDS.

5. Development of a research agenda to support the programme, including engagement of South African academic centres and research institutions.

6. Establishment of a robust system to monitor efficacy of the intervention, adverse drug events, resistance and improvement and coordination of patient information systems.

7. Development of staffing norms and standards for the delivery of antiretroviral therapy and assessment of human resource needs, including health system managers, clinicians, nurses, pharmacists, nutritionists and counsellors.

8. Creation of a programme management unit to coordinate the implementation of the programme and recommendations for its functions, structure, staffing and cost.

9. Development of a communications plan for health providers and the public, including what to expect from the proposed treatment programme.

10. Development of a detailed five-year programme budget and an estimated 10-year budget to implement the treatment programme.

11. Development of a detailed implementation schedule.

To be successfully implemented, the comprehensive plan needed significant additional investments in the public health system to improve its capacity, in particular, its human resource capacity. The comprehensive care and treatment plan was to be delivered in an integrated fashion within the public health system.

Yet more than half of the total expenditures envisaged in the plan were to go toward emphasizing prevention and promoting healthy lifestyles. In the absence of a cure for AIDS, effective prevention strategies are critical. These include provision of: barrier methods, voluntary counselling and HIV testing, PMTCT, post-exposure prophylaxis, syndromic management of sexually transmitted infections, TB management, and a large and sustained information, education and communication campaign.

The comprehensive plan proposed to build on testing programmes to diagnose HIV and measure disease progression so that proper care and treatment regimens could be implemented. That included: ongoing medical services to provide treatment for opportunistic infections associated with HIV and ultimately, the provision of ARVs to arrest the progression to AIDS; an extensive nutrition intervention; and programmes to integrate the provision of medical care with traditional methods of healing. A full range of community support services was also contemplated, including:, counselling; adherence support groups; community mobilization efforts to reduce stigma and discrimination; patient transport; home- and community-based care; and, when necessary, palliative care.

To take but one measure, Table 1 shows the anticipated patient demand for ARVs in the care programme by year, with HIV-positive patients undergoing periodic CD4 counts, and in those patients with CD4 counts of < 200 cells/mm^3^, the commencement of treatment with ARVs. The aim was to achieve universal (100%) treatment coverage of new AIDS cases by the end of 2007/08. The estimate was that just more than 1 million people would be on ARVs.

**Table 1:** South African National Department of Health Comprehensive Plan: planned number of patients on ARVs and associated costs and total costs

Year	New cases starting ARVs^a^	Total cases on ARVs^a^	Total ARV diagnostic costs (ZAR million)^b^	Total ARV drug costs (ZAR million)^c^	Total plan costs (ZAR million)^d^
2003/04	53,000	53,000	13	42	296
2004/05	138,315	188,665	108	369	1590
2005/06	215,689	381,177	227	725	2358
2006/07	299,516	645,740	394	1,118	3268
2007/08	411,889	1,001,534	620	1,650	4474

**Total**			**1362**	**3904**	**11,986**

**Source: ***Operational Plan for Comprehensive HIV and AIDS Care, Management and Treatment for South Africa*. Pretoria; National Department of Health, 19 November 2003.

Notes:

^a ^Table 16.8, p. 248

^b ^Table 16.11, p. 250

^c ^Table 16.13, p. 250

^d ^Table 16.20, p. 256

It was an ambitious plan, the likes of which had not been attempted before in a resource-challenged environment like South Africa. In total, 22,500 new health workers would be recruited over the five-year period, including 1,100 new doctors, who are critical to management of ARV treatment for AIDS-ill patients.

## Discussion and evaluation

### Available resources to achieve policy targets

The resources available over the period, 2000-2008, came from two sources: internal and external. The internal resources included national and provincial government contributions, as well as those of private individuals. The external funds came from three main sources: multilateral organizations, foreign governments, and private foundations and other non-governmental organizations. The major external contributors included the Global Fund to Fight AIDS, Tuberculosis and Malaria, the United States President's Emergency Plan for AIDS Relief (PEPFAR) and the European Union.

Table 2 indicates the extent of South African resources pledged to address the epidemic. It includes only government planned expenditure to achieve the aims of the comprehensive plan. This value needs to be tempered by the difficult-to-estimate level of private expenditure that occurred. The estimate provided here should be treated with caution. External partners have also contributed significantly, which has complemented local efforts. Since the adoption of the comprehensive plan, South Africa has committed a substantially increased level of domestic resources into the national AIDS response. Yet at its largest, it is only 0.16% of gross domestic product (GDP). Southern African neighbours with equally serious epidemics, but much less resources, have committed much larger per capita expenditures. In 2005, Botswana committed 2.07% of its GDP to HIV/AIDS-related expenditures, Malawi 4.16%, Zambia 2.79% and Zimbabwe 0.87% [4].

**Table 2:** Internal resources available for HIV/AIDS: South Africa, 2000-2008

	Year								
	2000	2001	2002	2003	2004	2005	2006	2007	2008
National government	-	-	-	352	453	396	433	456	970
National conditional grants to provinces	-	-	-	333	782	1135	1567	1646	1735
Provincial governments	-	-	-	284	365	514	874	1088	1225
Total (ZAR million)^a^	214	348	1000	970	1600	2045	2874	3190	3930
$/ZAR exchange rate^b^	6.96	8.62	10.53	7.57	6.48	6.38	6.79	7.07	8.30
**Total ($ million)**	**30.7**	**40.37**	**94.96**	**128.13**	**246.91**	**320.53**	**423.27**	**451.20**	**473.49**
GDP ($ billion)^c^	132.88	118.48	110.88	166.65	216.44	242.06	254.99	277.58	n/a
**SA government resources as a % of GDP**	**0.02**	**0.03**	**0.08**	**0.07**	**0.11**	**0.13**	**0.16**	**0.16**	n/a

**Sources: **See below.

Notes:

^a^National Treasury, **National and Provincial Budgets**. http://www.treasury.gov.za/documents/national%20budget/default.aspx for relevant years (Accessed 2 June 2009) and National Department of Health. Pretoria; National Department of Health. Values for 2000-2002 were only given in aggregate and not broken down by method of delivery.

^b^Oanda FXHistory: **Historical Currency Exchange Rates**. http://www.oanda.com/convert/fxhistory (Accessed 2 June 2009). The yearly value was calculated by taking the daily interbank rate for each day in each year and averaged.

^c^World Bank, **World Development Indicators**. http://web.worldbank.org/WBSITE/EXTERNAL/DATASTATISTICS/0,,contentMDK:21725423~pagePK:64133150~piPK:64133175~theSitePK:239419,00.html (Accessed 2 June 2009). South African GDP converted into $ equivalents.

### Internal sources

The system of governance in the South African federal system involves the national government primarily raises taxes and distributing these taxes to provinces in tied and untied grants for service delivery purposes. So national government controlled most of the available resources and overall policy direction for HIV/AIDS, but relied upon provincial governments to deliver services. Provincial government also engaged in discretionary spending on HIV/AIDS. The ambitious plan was projected to cost 11.986 billion South African rands, or US$1,915 million at prevailing exchange rates, over five years.

There are three main types of HIV and AIDS specific allocations. These are:

1. The budget of the HIV and AIDS Directorate in the national Department of Health (national equitable share).

2. HIV/AIDS interventions coming from national government to provinces (conditional grants).

3. HIV- and AIDS-specific funds in provincial budgets (equitable spend allocations).

Conditional grants are disbursements to provinces on the condition that they be spent on services or interventions specified by the national government. Spending of the funds is limited to specific areas identified by the national government for which provinces must develop appropriate business plans.

### Actual expenditure

#### Government

As shown in Table 3, expenditure up to and including 2003 concentrated largely on prevention activities, such as life-skills and HIV/AIDS training in primary and secondary schools, and free condom provision.

**Table 3:** Actual expenditure by HIV/AIDS programme: South Africa 2000-2008

	Year									
	2000	2001	2002	2003	2004	2005	2006	2007	2008	Total
Prevention	200	313	458	132	134	136	144	152	229	
Care & treatment	14	25	546							
Care				69	79	186	191	195	208	
Treatment				686	1235	1531	2001	2102	3078	
Total (ZAR million)	214^a^	348^a^	1004^a^	887^b^	1448^b^	1853^b^	2336^b^	2449^b^	2870^c^	13,846
$/ZAR exchange rate^d^	6.96	8.62	10.53	7.57	6.48	6.38	6.79	7.07	8.30	
**Total ($ million)**	**30.7**	**40.37**	**120.65**	**117.17**	**223.45**	**290.44**	**344.03**	**346.39**	**398.43**	**1,911.63**

**Sources: **See below.

Notes:

^a^Hickey A: **What Budget 2002 means for HIV/AIDS**. Budget Brief 90, IDASA Budget Information Service, February 26, 2002. www.idasa.org.za (Accessed 15 June 2009)

^b^Ndlovu N, **Budget allocations for HIV and AIDS in 2005/6 provincial sector budgets: Implications for Improved Spending**. Budget Brief 156, IDASA Budget Information Service, 5 August 2005. www.idasa.org.za (Accessed 15 June 2009)

^c^Mukotsanjera V, **HIV/AIDS domestic financing in South Africa**. February 2008, IDASA. www.idasa.org.za (Accessed 15 June 2009)

^d^Oanda FXHistory: **Historical Currency Exchange Rates**. http://www.oanda.com/convert/fxhistory (Accessed 15 June 2009). The yearly value was calculated by taking the daily interbank rate for each day in each year and averaged.

Over the five years of the comprehensive plan, actual government spending totalled $1,602 million as opposed to projected budgeted spending of $1,915 million. This represents an under spend of $313 million. The main reasons for the under spend was the performance of provinces in being unable to implement the required health system changes in a timely manner and an inability to hire sufficient health workers to enable the ambitious programme goals to be achieved.

#### Private individuals

South Africa has an extensive and sophisicated private health care system. The comprehensive plan did not incorporate nor attempt to engage with the private health system.

#### External sources

The Global Fund, established in 2002, is a partnership between governments, civil society, the private sector and affected communities. It has become the main external source of finance for programmes to fight AIDS, tuberculosis and malaria, with approved funding of US$11.4 billion for more than 550 programmes in 136 countries. It provides a quarter of all international financing for AIDS globally. The Global Fund's contributions to South Africa since 2003 have totalled $228.5 million. This amount has been channelled through the national government budget, so it has inflated the supposed contribution of the national government to internal funding towards HIV/AIDS.

The largest single increase in external funding for HIV/AIDS came from the US Government via President George Bush's 2003 initiative, PEPFAR, as shown in Table 4. Total funding pledged for the first five fiscal years was US$15 billion, although a total of $18.8 billion was expended in 2004-2008. As part of the PEPFAR contribution, the US Government has pledged $4 billion out of a total Global Fund pledge of $18 billion in the fiscal years of 2002 to 2008.

**Table 4:** US Government resources for HIV/AIDS: South Africa 2003-2008 ($ million)

Year	2004	2005	2006	2007	2008	Total
**Value**	**89.3**	**148.2**	**221.5**	**397.8**	**590.9**	**1,447.7**

**Source: **The United States President's Emergency Plan for AIDS Relief (PEPFAR), **Country Operational Plan Summaries - South Africa**. Various years. http://www.pepfar.gov/countries/c19712.htm (Accessed 15 June 2009)

South Africa is one of PEPFAR's 15 focus countries, which collectively represent approximately 50% of HIV infections worldwide. Under PEPFAR, South Africa received $89.3 million in fiscal year (FY) 2004, $148.2 million in FY 2005, $221.5 million in FY 2006, $397.8 million in FY 2007 and $590.9 million in FY 2008 to support comprehensive HIV/AIDS prevention, treatment and care programmes. This is a total of $1,447 million over the past five years.

### Treatment data

Objective criteria provide evidence with which an evaluation of the effectiveness of responses within countries can be made. In the case of South Africa's comprehensive plan, these data indicate that the responses to ameliorate the epidemic have been only partially effective.

Given that the majority of HIV-infected individuals interact with the public health sector, then an examination of the progress in the biggest HIV-related programme, the National ARV Treatment Programme, should be instructive.

There are no accurate estimates of the total number of people on antiretroviral treatment because of such factors as the national Department of Health's poor monitoring system (except for the Western Cape province), no province records, loss to follow up and deaths.

The Joint Civil Society Monitoring Forum was formed in June 2004 and is made up of several leading civil society and private sector organizations. The forum is dedicated to monitoring the implementation of the operational plan. Its latest estimate is that in June 2007, a total of 257,108 people were on treatment [5].

This contrasts with the Department of Health's assertion that that the estimated number of people needing treatment in South Africa was 764,000 by the middle of 2006, of which a total of 353,945 were enrolled in the ART programme and 273,400 were initiated on the programme in 2006. In 2007, 889,000 people needed treatment, of whom 488,739 enrolled and 371,731 were initiated on the ART programme [6]. These statistics were derived from a statistic model as opposed to actual clinic data, and they should be treated with great caution.

The World Health Organization's and UNAIDS' midpoint estimate was that 206,500 people living with HIV (PLHIV), or equivalent to 21% of the number estimated to be in need, were on ART in South Africa as at December 2005. These values should be treated with caution given the South African Government data upon which the estimate was made [7].

An estimate by a major supplier (Aspen Pharmacare) of ARVs delivered to the Department of Health is that about 350,000 people were on treatment as at February 2008 [8]. Aspen supplies 80% of the public sector's ARV drug, lamivudine. Nearly all first-line patients are put on lamivudine. Apparently, the company projected its sales to the public sector and then added on the remaining supply of lamivudine by GlaxoSmithKline and a projection for the number of people who have moved to second-line therapy. The calculation is not in the public domain and should be treated with great caution.

No comprehensive methodical analysis of the number of people on ARVs in the private health system has been done. The Joint Civil Society Monitoring Forum estimates that in the order of 100,000 people were receiving treatment through the private sector in 2007 [5]. This estimate should be treated with some caution.

### Estimate of number of untreated lives lost

Whatever the accurate numbers are, the uptake of ARVs has fallen well short of anticipated levels. Many hundreds of thousands on South Africans in need of ARVs are still not receiving them or have died whilst waiting for them, despite the comprehensive plan.

An estimate of the loss of life years that resulted from the ineffectual policy responses of President Mbeki and Minister Tshabalala-Msimang was recently made. The study compared the number of persons who received ARVs for treatment and PMTCT transmission between 2000 and 2005 with an alternative of what was reasonably feasible in the country during that period. It was estimated that more than 330,000 lives, or approximately 2.2 million life years, were lost because a timely ARV treatment programme was not implemented in South Africa over that period. Some 35,000 babies were born with HIV, resulting in 1.6 million life years lost by not implementing a PMTCT programme using the ARV drug, nevirapine. The total lost benefits of ARVs not being accessible to all in need are estimated at 3.8 million life years for the period, 2000-2005 [9].

### Value of lives lost

The value of a human life or one additional year of a human life is inherently controversial. The benefit of the provision of ARVs is that it stops PLHIV dying prematurely. It also has another advantage in that it generally improves the quality of life of those years gained.

The above estimate of life years lost does not take into account the value of those life years to society. Given that the costs of treating and also of not treating PLHIV with ARVs has been made and an estimate of the number of life years has also been made, then it is logical to attempt to value the benefits that would have accrued to South African society if those lives and life years had not been lost.

There are two main methods used in measuring the value of a human life: human capital approach and willingness to pay (WTP) [10,11]. Both are controversial and have many methodological difficulties. Historically, the first attempts at valuing lives saved used the human capital approach. In this approach, a human being is regarded as an asset with a capital value based (as is the case for any asset) on the future returns it will earn. In the human case, these returns are earnings. Hence, the value of a life is the present value of the stream of expected future earnings.

Some implications are that the young are generally worth more than the old, although the very young, who have yet to incur education and upbringing costs, may have quite a low value. High-income individuals have a greater value than the poor. Questions that arise in using the human capital approach are whether gross or net earnings, or gross earnings less consumption, should be used. The arguments are that although with death an individual ceases to be a member of society (hence their death is costless *per se*), it is his or her contribution to the rest of society which is lost and should be valued.

The main criticisms of this approach are:

• Non-productive individuals (e.g., the elderly and chronically ill) have negative returns so that any lengthening of their lives represents a loss.

• Consumption benefits of health care are not included.

• Earnings do not reflect social productivity because if an individual dies, his or her position would be filled by someone else so that the loss of production will be related to adjustments necessary, not the earnings of the replaced individual.

The other main approach is based on willingness to pay (WTP) to reduce the risk of death. Health care projects that save lives do not (except in the very short term) save specific lives, but rather reduce the risks faced by all or a subset of the population. Hence, it may be possible, by asking or by carrying out appropriate experiments, to ascertain the WTP of relevant groups for a reduction in the risk of death from a particular cause.

Related approaches use market prices to infer the value individuals place on reductions in risk. For example, the amounts individuals spend on life-protecting safety devices or on safer forms of transport may be used to infer valuations. Another approach is based on occupational risk: the argument is that measurement of the monetary compensation (higher wages) received for high-risk occupations will allow us to infer valuations [11]. If an individual earns an extra $10,000 per annum for facing an increase of 20 percentage points in the chance of dying each year, then it may be inferred that the individual values their life at $50,000 per annum. Taking the present value of this stream of values will give the capital value.

Besides saving lives, health care offers benefits of many kinds. It may reduce pain and discomfort, increase mobility and generate peace of mind. How might these various effects be valued?

The approaches taken to answering this question mirror to a large extent the approaches taken to the valuation of life. A human capital approach would find the present value of the difference in lifetime earnings between those receiving and those not receiving treatment. The assumption behind this is that all the adverse effects of a medical problem will show up in earnings. A WTP approach would try to find out, by asking or by experiment, what individuals are prepared to pay to avoid the effects of a particular condition or to reduce the risks of suffering these effects. Observation of market behaviour (e.g., of amounts spent on medicines) may allow inferences to be drawn about WTP to avert certain types of effects. Given a societal perspective, there is a maximum WTP that represents the benefits (utility) that all individuals would expect to achieve if they had access to a particular health care service.

Next, it is necessary to assess the resources required to provide that service (cost) and to compare this to the total value to society (benefit) from having access to a health care service. In the societal perspective, the benefits of improved health care are comprised of the benefits to the affected individuals (use value), as well as the benefits that the rest of society can expect to achieve from knowing that the service is available (non-use value).

One objective may be to determine the value to an individual or community of a particular good or service. Then it is necessary to evaluate the value of the service to all of those who may benefit from this service (both users and non-users) and to compare this to the costs. If an individual or a community agree that they want to give up their financial resources to pay for a good or service (through user charges, private insurance, taxes, social insurance or charities), then it can be concluded that the benefits exceed the costs. The benefit-cost ratio is greater than one and the intervention is recommended. However, if individuals or a community indicate that they are unwilling or unable to give up the financial resources to provide a health service, then the costs exceed the benefits. If the service has a benefit-cost ratio of less than one, then intervention is not recommended.

To overcome philosophical objections and methodological difficulties in valuing life and life years, it has been assumed here that the value of one year of human life is equivalent to the value of per capita South Africa's GDP in the year of that human life. That is, the value of one year of human life is equivalent to the value of economic output for an average South African during one calendar year.

The economic cost of those 3.8 million lost life years over the period, 2000 to 2005, has been calculated in Table 5. Conservatively assuming that the value of one life year is equivalent to the per capita contribution to GDP in that year, it is estimated that the economic cost to South African society of these avoidable life years lost through premature death over the six-year period is more than $15 billion.

**Table 5:** Value of HIV/AIDS lives lost: South Africa 2000-2005 ($)

Year	Lost life years - ART^a^	Lost life years - PMTCT^a^	Total lost life years^a^	GDP per capita US$^b^	Lost annual GDP $^c^	As a % of GDP^d^
2000	36,180	25,380	61,560	3042	187,539,300	0.001
2001	126,630	152,280	278,910	2644	737,438,040	0.006
2002	330,310	279,180	609,490	2439	1,486,546,110	0.013
2003	524,610	380,700	905,310	3589	3,249,157,590	0.019
2004	643,200	444,150	1,087,350	4646	5,051,828,100	0.023
2005	578,880	317,250	896,130	5163	4,626,719,190	0.019

**Total**	**2,239,810**	**1,598,940**	**3,838,750**		**15,339,228,000**	
					**$15.33 billion**	

**Sources: **see below.

Notes:

^a^Chigwedere P, *et al*: **Estimating the Lost Benefits of Antiretroviral Drug Use in South Africa**. *Journal of Acquired Immune Deficiency Syndrome *2008 **44**:410-415. Tables 1 and 2.

^b^Author's calculation. GDP values obtained from World Bank, **World Development Indicators**. http://web.worldbank.org/WBSITE/EXTERNAL/DATASTATISTICS/0,,contentMDK:21725423~pagePK:64133150~piPK:64133175~theSitePK:239419,00.html (Accessed 15 June 2009). South African GDP converted into $ equivalents. Population values obtained from Statistics South Africa, **Mid-Year Population Estimates**. Publication P0302 - Various Years. www.statssa.gov.za/publications/p0302/populationstats.asp (Accessed 15 June 2009)

^c^Author's calculation.

^d^Author's calculation.

Given that the actual expenditure by government on HIV/AIDS programmes over the same period was $822.78 million (from Table 3), it would seem the orders of magnitude would strongly suggest that higher levels of expenditure should have been made to avoid the extremely large reduction in GDP of $15 billion, which arose as a result of the inadequate treatment response by government.

### Reasons for lack of policy effectiveness

Early on in the epidemic, Jonathan Mann outlined a three-point typology for describing the policy response to epidemics of infectious or communicable diseases like HIV/AIDS. The three stages through which policy responses can move forward, and unfortunately sometimes backwards, are [12]:

• First: Denial - that the epidemic is present within the country, reflected either by an absence of any preventative or treatment measures or by entry restrictions for foreigners with HIV.

• Second: Recognition - that the epidemic is present in the country. A country will admit that cases of the epidemic are occurring and will adopt measures to find out how widespread the epidemic is.

• Third: Mobilization - will finally occur, which means that a country gets active, both on societal and government level to hinder the further spread of the epidemic.

HIV/AIDS received scant attention from the National Party government prior to 1994. It was not seen as a major problem or, if perceived as such, it was seen as a "black man's disease". The new democratic government had many issues confronting it and HIV/AIDS did not rank very highly given its (then) relative insignificance. Yet a discernable shift from denial to recognition of the epidemic was seen in President Mandela's public references to the issue, unlike his predecessors. Unfortunately, the level of infection was rapidly growing. In 1993, the HIV prevalence rate among pregnant women was 4.3%, which had increased to 12.2% by 1996 and to 22.4% by 1998 [13].

In 1999, newly installed President Mbeki stated that the drug, AZT, used in the prevention of mother to child transmission treatment (PMTCT), was toxic and dangerous to health and that the government would not be provide it in the public health system [14]. He went further and defended a small group of dissident scientists who claim that AIDS is not caused by HIV, and questioned the efficacy of all antiretroviral drugs because they target HIV [15].

In April 2000, in his opening speech to the International AIDS Conference in Durban, President Mbeki avoided reference to HIV and instead focused on the problem of poverty, fuelling suspicions that he saw poverty, rather than HIV, as the main cause of AIDS. The basis for President Mbeki's denialism and reversal of government policy positions were never clearly enunciated by the man himself, although Nattrass discusses the many and varied hypotheses, clearly favouring the President's belief in his exceptionally high option of his intellectual capacities which he believed outranked those of medical and social "experts" in the field [16,17]

There was now clear evidence that the South African Government was moving from recognition back to denial about the epidemic. At that time, most political leaders in sub-Saharan Africa would have been considered to be in a state of denial, but in the process of moving toward recognition as evidence of the impacts of the epidemic were becoming increasingly hard to avoid [18]. President Mbeki and his administration were moving in the opposite direction. Minister Tshabalala-Msimang had responsibility for health policy, including HIV/AIDS. She and President Mbeki were repeatedly accused of failing to respond adequately to the epidemic. Fortunately, the professionals in the under-resourced public health system in South Africa attempted to respond to the treatment needs of HIV-positive people for opportunistic infections, although these systems were overwhelmed by the scale of need and the lack of antiretroviral drugs.

Yet there were also signs of hope. President Mbeki's government was applauded by AIDS activists for its successful legal defence against action brought by multinational pharmaceutical companies in April 2001 of a law that would allow local production of cheaper medicines. Initial prices of ARV drugs were extremely high for a middle-income country like South Africa. It was only in 2002 and 2003 that prices began to moderate sufficiently to allow low-income countries to seriously consider universal treatment options. People in South Africa obtain medicines either through the public health system or from private dispensing doctors and pharmacies. Patients receive medicines for free from the public health system, but have to put up with long waiting times and inconsistent and missing service. Private sector patients are usually insured by a medical scheme to which they pay a monthly premium.

In 2002, South Africa's High Court ordered the government to make the ARV drug, nevirapine, available to pregnant women to help prevent mother to child transmission of HIV. Despite international drug companies offering free or cheap antiretroviral drugs, President Mbeki's government restricted access to them and remained extremely hesitant about providing treatment for people living with HIV.

Despite the AIDS denialism of President Mbeki and Minister Tshabalala-Msimang, significant government financial resources were mobilized. However, these resources were insufficient despite the efforts of the South African Government and the initiation of PEPFAR after the announcement of the comprehensive plan. Less than half of the people requiring ARVs are currently receiving them. Some of the reasons for this lack of effectiveness are now briefly discussed.

Possible explanations for the slow and ineffective responses include:

#### Political commitment

"Positive" political discussion and action about HIV is relatively scarce in South Africa. Successful country responses to the epidemic, as in Uganda and Senegal, have had in common the existence of political will or commitment from the head of state downwards. Stigma and discrimination lie behind the denial and silence from South African political leaders. As long as HIV is not discussed openly, denial of the problem will exist. The importance of leaders in addressing HIV to overcome silence and stigma is critical. Without "positive" political dialogue, the problems that arise from HIV infection will continue to be surrounded by ignorance, myths and, of course, denial that the problem exists in the first place.

The effort of government toward implementing the comprehensive plan was damaged by the attitude towards HIV/AIDS and its treatment by Minister Tshabalala-Msimang and President Mbeki. Tshabalala-Msimang's administration as Minister of Health was controversial because of her reluctance to adopt a public sector plan for treating AIDS with ARVs. She was called "Dr Beetroot" for promoting the benefits of beetroot, garlic and lemons, as well as focusing on good general nutrition, while referring to possible toxicities of ARVs. She followed an AIDS policy in line with the ideas of President Mbeki, her political ally.

Minister Tshabalala-Msimang placed her emphasis on broad public health goals, seeing AIDS as only one aspect of that effort and one which, because of the financial costs of treatment, might impede broader efforts. She was not convinced by the mounting economic evidence that AIDS is such a burden on the public health system that treating it would actually free up costs. She was in charge of the ARV rollout, but continued to emphasize the importance of nutrition and to urge others to see AIDS as only one problem among many in South African health.

At the International AIDS Conference in Toronto, Canada, on 18 August 2006, Stephen Lewis, the United Nations Special Envoy for AIDS in Africa, closed the conference with a sharp critique of South Africa's government. He said South Africa promoted a "lunatic fringe" attitude toward HIV and AIDS, describing the government as "obtuse, dilatory, and negligent about rolling out treatment" [19].

#### Health system capabilities (especially human resources for health)

A lack of resources, both physical and human, to improve the health of the population existed prior to the comprehensive plan. The infrastructure required for the increased levels of health activities was often lacking. Hospitals, clinics, health staff and consumables were commonly in short supply before the epidemic. The epidemic merely places more pressure on resources despite additional funding being available.

There are insufficient health workers in South Africa to enable normal health care needs to be met. The maldistribution and inadequate numbers of health workers are causing delays in expanding ARV treatment. Waiting lists for treatment are growing at clinics as a result of staff shortages. Even with "task shifting" from doctors to nurses to community workers, insufficient staff means that ARV diagnosis, treatment and monitoring is restricted. It has recently been suggested that an extension of this community-based pathway is possibly the only means by which the increasing ARV case loads can be adequately managed [20].

There is a general shortage of health workers in South Africa. The shortage is clear in the number of vacant public health worker posts, which show that out of a required workforce of 196,585, 65,432 posts were unfilled [21].

This shortage is further exacerbated by the highly uneven distribution of health workers between the public and private sectors. The ratios of medical practitioners to population in public and private sector are, respectively, one per 4,219 and one per 602 [21]. The comprehensive plan utilized only public health workers. This unevenness is also shown in the geographical distribution, with rural areas having a much lower ratio of health workers to population than urban areas.

The private health sector in South Africa is highly formalized, well developed and resource intensive. Health professionals are attracted from the public to the private sector by higher remuneration rates, more favourable working conditions and better access to advanced technology [22].

In addition to facing shortages of staff throughout the public health system, South Africa faces additional challenges in retaining health workers due to increasing levels of migration. In 2006, the number of South African health workers working abroad totalled 23,400: 8900 doctors, 6800 nurses and 7700 other health workers [21].

Given the skills shortage in health care, the number of new graduates produced annually is a possible key area for intervention. In South Africa, there are 401 nursing education institutions and eight medical schools. The average number of enrolments per medical school per annum was 200 in 2007, which equates to approximately 1600 enrolments nationally [21]. Doctors take a minimum of six years to train, and are required to do one year of community service before being allowed to work in the private sector.

There are three main streams through which nurses are trained: universities, nurse training colleges and two-year bridging courses. The bridging courses enable enrolled nurses and nursing auxiliaries to train and register as professional nurses. In 2006, the total number of nurses graduating from universities and training colleges totalled 2027 [21].

Clearly, the mobilization of the private sector is critical to achieving the goals of the comprehensive plan. Present numbers of health workers are insufficient and present trends for training new and replacement workers are clearly inadequate. Until the issue of resource mobilization in health, especially human resources, is adequately addressed, the goals of the comprehensive plan will prove difficult to achieve.

## Conclusions

Progress in addressing the HIV/AIDS epidemic has been made in South Africa, which is one of the few countries in sub-Saharan Africa with the resources to provide ART for all of its people with AIDS. However, the majority of patients who require ART are still not receiving it. As a result, in most hospitals in South Africa, it is still common to see patients without access to ART dying of opportunistic infections, including TB. There were an estimated 360,000 AIDS deaths in 2007. This has brought the cumulative number of AIDS deaths to 2.2 million people [1].

The estimated number of deaths as a result of an inadequate policy response between 2000 and 2005 was that more than 330,000 lives, or approximately 2.2 million life years, were lost because a timely ARV treatment programme was not implemented in South Africa over that period. Furthermore, 35,000 babies were born with HIV, resulting in 1.6 million life years lost by not implementing a PMTCT programme using the ARV drug, nevirapine. The total lost benefits of ARVs are estimated at 3.8 million life years for the period, 2000-2005 [16].

The economic cost of those 3.8 million lost life years over the period, 2000 to 2005, through premature death over the six-year period is more than $15 billion.

This paper has attempted to explain HIV/AIDS policy responses and the resources available to achieve the goals over the period, 1999 to 2008. An explanation of the reasons behind the failure to implement the national ART programme in a timely and effective manner from 2003 onwards is offered, as is how the recent progress towards universal ART coverage might be improved and/or achieved.

A new five-year National Strategic AIDS Plan 2007-2011 [23] has been introduced, and in August 2008, the removal of President Mbeki and simultaneously the replacement of Minister Tshabalala-Msimang with a new Minister for Health energized AIDS activism in South Africa. The new plan allocates about R45 billion (about $6 billion) towards HIV/AIDS prevention and treatment.

## Competing interests

The author declares that he has no competing interests.

## Authors' contributions

JG conceived the study and its design, undertook the analysis, and wrote the manuscript.
